# Investigating the effects of acute caffeine administration on visual search, resistance to distraction, and attentional learning

**DOI:** 10.1186/s41235-026-00760-8

**Published:** 2026-10-21

**Authors:** Sojung Youn, Jenna Glotfelty, Ming-Ray Liao, Brian A. Anderson

**Affiliations:** Department of Psychological and Brain Sciences, Texas A & M University, College Station, TX 77843 USA

**Keywords:** Caffeine, Visual search, Selective attention, Statistical learning

## Abstract

The ability to selectively attend to task-relevant information is central to the performance of a variety of cognitive tasks. Accessible and low-risk pharmacological interventions hold promise with respect to maximizing cognitive performance and augmenting attentional abilities. Acute administration of caffeine reflects one such pharmacological intervention, which has been shown to improve some measures of attention. The effects of caffeine on the efficiency of attentional selection during visual search and the degree to which such efficiency can improve with learning have not been investigated, which we tested here. In a single-blind between-subjects design, participants received either 200 mg of caffeine or placebo and completed visual search tasks in which the target (Experiment 1) and a physically salient distractor (Experiment 2) were more likely to be rendered in a particular color. A third task was also implemented to measure the trade-off between physical and attentional effort under the effect of caffeine (Experiment 3). The efficiency of target selection (Experiment 1) and distractor rejection (Experiment 2) were strongly influenced by color-based statistical learning. Participants were sensitive to the trade-off between physical and attentional effort (Experiment 3). However, none of these measures were influenced by caffeine. A beneficial effect of caffeine was observed on the speed of target recognition in Experiment 1 only. Our findings suggest a limited role for the influence of caffeine on the attention system.

## Significance statement

Caffeine, a substance that is deeply embedded in our daily lives, is colloquially believed to serve as a “cognitive enhancer” with beneficial effects on attention. Despite this popular belief, we know little about the effect of caffeine on attention. Attention is a critical cognitive process that allows individuals to prioritize relevant information while filtering out distractions. The current study offers insight into the effect of caffeine on attention through a series of experiments that probe attentional selection of relevant information, the suppression of irrelevant distractions, and the mental effort involved in controlling attention. By clarifying how caffeine influences attention, this research advances our basic understanding of cognitive control while also informing practical decisions about its use in education, work, and safety–critical tasks. In the present study, caffeine had a limited impact on attention across the three experiments, casting doubt on the effectiveness of caffeine as a pharmacological means of broadly enhancing attentional performance.

## Introduction

In most environments encountered in everyday life, the visual world presents more information than a person can process at any one moment in time. Objects compete for representation in the brain, and attention serves as the mechanism by which one of multiple sensory inputs is selectively processed in perceptual systems (Desimone & Duncan, 1995). Adaptive, situated behavior requires selectively attending to pertinent information and efficiently ignoring contextually irrelevant information (e.g., Anderson, 2021). The ability to selectively allocate visual attention therefore reflects a potentially high-impact target for intervention, with improved attentional control having potentially broad benefits for maximizing human performance.

Self-administration of substances believed to have cognition-enhancing properties has become increasingly prevalent, including the off-label use of prescription drugs (Sharif et al., 2021). Caffeine is a non-prescription drug that is broadly used for its alerting properties, but research suggests that acute caffeine administration may have cognitive benefits as well. As a cognitive process, attention can be decomposed into orienting, alerting, and executive components (Fan et al., 2002, 2005); each of these components can be further differentiated, for example by target selection and distractor filtering in the case of orienting (e.g., Theeuwes, 2010; Wolfe et al., 2020, 2021) and phasic alerting to a cue versus sustained attention in the case of alerting (e.g., Esterman & Rothlein, 2019; Esterman et al., 2013; Fan et al., 2002, 2005). Processes impacting attentional orienting can be further defined as voluntary/goal-directed and involuntary or stimulus-driven (Liesefeld et al., 2024). Understanding the impact of caffeine on cognition requires consideration of these different component processes within the domain of attention and how each may be influenced by the substance.

Identification of a target letter among non-targets letters (i.e., distractors) during a spatial attention task can be sped up by caffeine, which was also reflected in enhanced electroencephalographic measures of selective attentional processing (Lorist et al., 1994, 1996). Using the Attention Networks Test (ANT) with arrow flankers, the alerting and executive components of attention can be improved with caffeine (Brunyé et al., 2010a, 2010b). Stroop interference, taxing executive attention, has also been shown to be reduced by acute caffeine administration (Kenemans et al., 1999; see also van den Berg et al., 2021). While caffeine has been shown to speed responses across a variety of tasks (e.g., Kenemans et al., 1998; Lorist & Snel, 1997; Lorist et al., 1996; Ruijter et al., 1999; Wilhelmus et al., 2017), this speeding appears to be more strongly mediated by attentional than motoric processes within tasks that probe sustained attention (Saville et al., 2018).

However, some studies have failed to observe an effect of caffeine on the selectivity of information processing (e.g., Kenemans et al., 1998; Nehlig, 2010). There is also some evidence to suggest that effects of caffeine on attentional performance may be explained, at least in part, by a more general influence of arousal rather than an enhancement of cognitive processes. A visual search task with differential memory load (e.g., 2- and 6-letter) showed an effect of caffeine administration; however its influence was better explained by an effect on arousal, with increased search accuracy in the lower memory load task and reduced accuracy in the higher memory load task (Anderson & Revelle, 1983). Another study showed no evidence for an interaction between caffeine and task difficulty or the degree to which the task relies on automaticity (consistent vs. variable mapping; Loke, 1992), further suggesting that the control of attention may not itself be improved through the administration of caffeine. Whether and how self-administration of caffeine should be expected to impact attentional performance remains to be clarified.

In the present study, we examined the influence of caffeine on multiple indicators of selective attention in the context of visual search, which have been less emphasized by prior research on the effects of caffeine. Experiment 1 utilized a spatial configuration search task in which participants must localize and report a target “T” among L-shaped distractors (see Duncan & Humphreys, 1989; Jiang et al., 2013; Wolfe, 1998). Importantly, the number of L-shaped distractors varies, permitting a measurement of the efficiency with which non-targets can be rejected (distractor filtering, reflected in search slope) and the speed with which the target can be identified and reported (search intercept), the latter of which involves the detection of a match between an attended stimulus and a target template held in memory (Wolfe, 2020; Woodman et al., 2013). Experiment 2 utilized an additional singleton task in which participants search for and report a uniquely shaped target presented among non-target items, one of which is sometimes rendered in a unique color (Theeuwes, 1992, 2010). Of interest in this task is the performance cost associated with the presence of the uniquely colored (and therefore physically salient) distractor, which is attributable to involuntary distraction by this task-irrelevant stimulus (termed *attentional capture*; Liesefeld et al., 2024). The efficiency of voluntary attentional control in the context of visual search is a critical component of human information processing (e.g., Wolfe, 2020, 2021) and difficulty managing distraction can have a variety of detrimental consequences both with respect to productivity and health/safety (see Anderson, 2024; Anderson et al., 2024). However, search efficiency and susceptibility to distraction have not been investigated in the context of acute caffeine administration.

The efficiency of attentional selection can also improve as a function of task-specific learning processes. Referred to as *selection history* (see Awh et al., 2012; Anderson et al., 2021), past episodes of attentional selection in particular task contexts can facilitate the future allocation of attention in similar contexts. Through selection history, observers can learn to more efficiently select a repeated target (e.g., Anderson & Britton, 2019; Jiang et al., 2013; Kim & Anderson, 2019; Kyllingsbaek et al., 2001; Shiffrin & Schneider, 1977) and more efficiently reject a frequently encountered distractor (Kim & Anderson, 2022; Kim et al., 2025; Stilwell et al., 2019; Theeuwes et al., 2022; Wang & Theeuwes, 2018a, 2018b, 2018c), in some cases to the point of near-complete ignoring (see Anderson et al., 2024). Outside of the context of attention, caffeine has been shown to promote learning and memory under certain conditions, both with respect to incidental learning (see Nehlig, 2010, for a review) and post-encoding consolidation (Borota et al., 2014). In intentional learning contexts (see Nehlig, 2010, for a review) and at the time of retrieval (Borota et al., 2014), caffeine generally does not benefit learning and memory. The influence of selection history on attention is largely implicit and, in some cases, can proceed without awareness (e.g., Grégoire & Anderson, 2019; Grégoire et al., 2022; Jiang et al., 2013; Leganes-Fonteneau et al., 2018, 2019; Wang & Theeuwes, 2018a). Such learning-dependent attentional processes could therefore also be subject to augmentation through acute caffeine administration, which we additionally explore in the present study through manipulations of selection history in the context of both the facilitation of target selection (Experiment 1) and the efficiency with which a salient but irrelevant stimulus can be ignored (Experiment 2).

## Experiment 1

Experiment 1 involved a visual search task very similar to the one used by Anderson and Lee (2023) but with the physical effort component removed. On each trial, a sideways “T” was presented among L-shape distractors and participants needed to report whether the top of the “T” was leftward or rightward facing. The search items were presented in different colors, one of which was more likely to serve as the color of the target on a given trial. To the degree that participants are sensitive to the color statistics of the task, they should be faster to find the target when it is rendered in the high-probability color, consistent with a prior report (Anderson, 2026). The number of items in the search display varied between 5 and 50 across trials, permitting a calculation of the influence of adding non-targets (search slope) and the process of identifying and reporting the target (search intercept) on response time. Half of the participants received 200 mg of caffeine via dissolvable strips while the other half of participants received dissolvable breath strips that did not contain caffeine (placebo). We compared the search slope and intercept between the caffeine and placebo groups, separately for trials on which the target was and was not rendered in the high-probability color.

### Method

#### Participants

Ninety-one participants between the ages of 18 and 35 years inclusive, 45 in the caffeine group (27 females, 18 males, *M*_age_ = 19.09, SD = 1.06) and 46 in the placebo group (31 females, 15 males, *M*_age_ = 19.09, SD = 0.81), were recruited from the Texas A&M University community. All participants were English-speaking and reported normal or corrected-to-normal visual acuity and normal color vision. Participants self-reported abstaining from caffeine intake for a minimum of four hours prior to their study appointment and regularly consuming less than five shots of espresso worth of caffeine per day. To be eligible, participants needed to report having previously consumed caffeine without adverse reaction, although beyond that, there was no requirement concerning minimal caffeine intake. Participants were undergraduate students who were compensated with course credit. All procedures were approved by the Texas A&M Institutional Review Board. Written informed consent was obtained for each participant and all study procedures were conducted in accordance with the principles expressed in the Declaration of Helsinki.

A target sample size of *n* = 45 per group was determined a priori to yield power (1-β) = 0.8 with α = 0.05 to detect a difference in search slope or intercept between groups, via direct comparison of means, of a moderate effect size *d* = 0.6, and a difference between the frequent and infrequent color conditions of a small effect size *d*_*z*_ = 0.3; scheduling budgeted for no-shows and resulted in an additional participant being obtained for the placebo group. The acquired sample also yielded power (1-β) = 0.8 to replicate the between-subjects main effect of caffeine on the Shifting Attention Task reported in Konishi et al. (2018) using a 2×2 mixed-effects design as in the present study (ANOVA repeated measures: between factors, *f* = 0.26 with two groups and two measurements per group, α = 0.05, correlation among repeating measures = 0.5 [default]). The smallest between-subjects effect that could be detected with power (1-β) ≥ 0.8 in a 2×2 mixed design was *f* = 0.257. All power analyses were computed using G*Power 3.1.

#### Apparatus

A Dell OptiPlex 7040 equipped computer with MATLAB software and Psychophysics Toolbox extensions (Brainard, 1997) was used to present the stimuli on a Dell P2717H monitor. Responses were entered using a standard US-layout keyboard. The participants viewed the monitor from a distance of approximately 70 cm in a dimly lit room.

#### Stimuli and materials

**Fig. 1:**
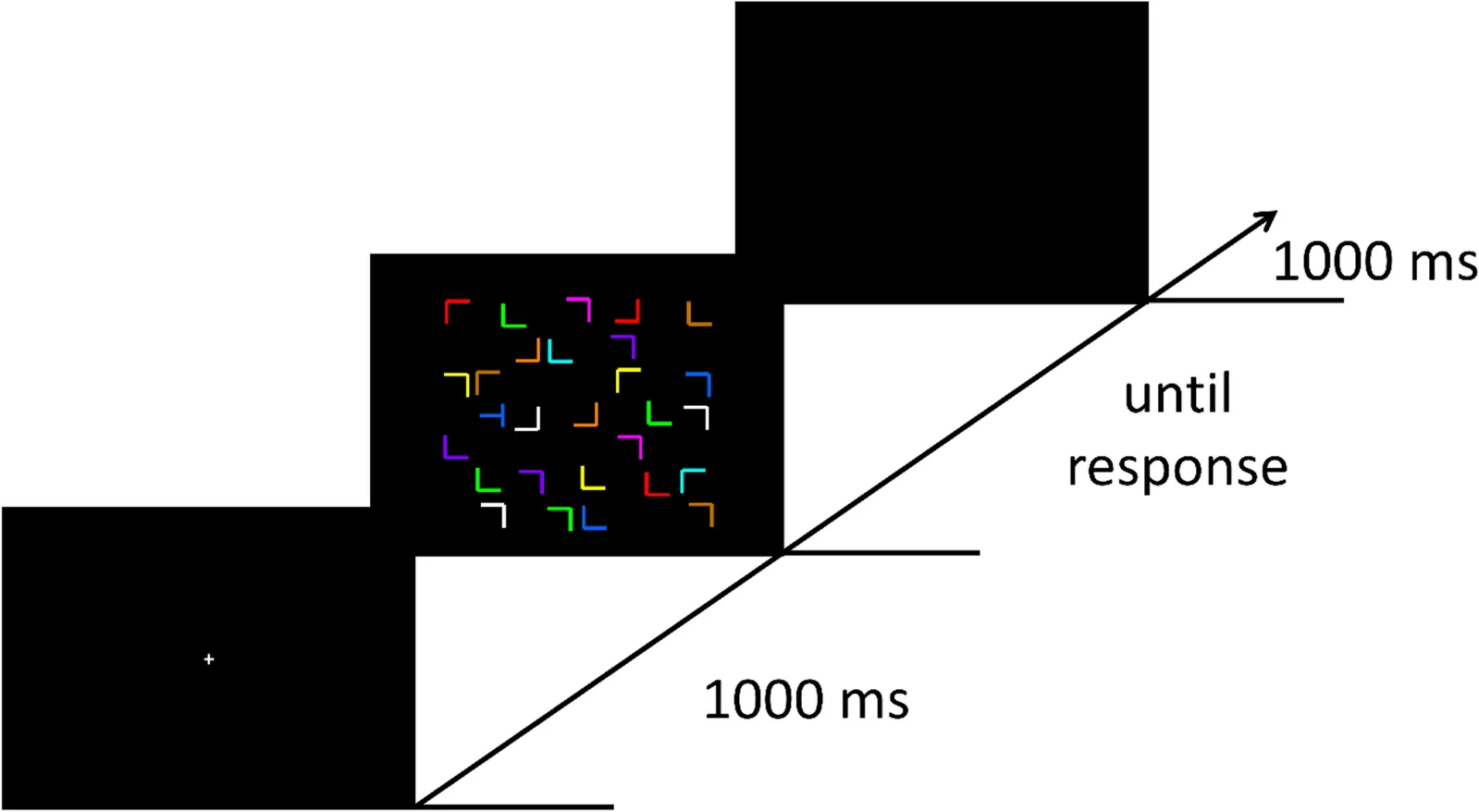
Example trial with time course of events for Experiment 1

Each trial consisted of a fixation display, a search display, and an inter-trial-interval (ITI). All stimuli were presented against a black background. The fixation display consisted of a white plus-sign (0.7° × 0.7°) presented at the center of the screen (see Fig. 1). The search display consisted of between 5 and 50 letters (each 1.5° × 1.5°) presented in an 8×7 grid (neighboring grid positions were separated by 3.6° in each dimension). One of these letters was a sideways “T” and the others were “L” shaped distractors in four possible orientations. The exact position of each letter was jittered by up to 0.7° both vertically and horizontally in either direction from the center of its respective grid position, creating an irregular grid. The colors of the letter stimuli could be red, green, blue, yellow, white, purple, pink, brown, orange, and cyan. The ITI consisted of a blank screen if the correct response to the target was made, whereas the word “Incorrect” was added to the center of the screen if the wrong response had been made in target identification.

Caffeine was administered sublingually via two 100 mg dissolvable strips (Revvies Extra Strength Energy Strips, Cola Lemon flavor). Placebo consisted of two dissolvable breath strips (Listerine Freshburst, mint flavor). The sublingual administration of caffeine allows for substantially faster absorption and effect than ingested caffeine.

#### Design

The experiment consisted of 10 blocks of 60 trials each (600 trials total). The number of letters in the search display varied from five to 50 in increments of five, with each set size used equally often in each block. The grid position occupied by the target was randomly selected on each trial, as were the positions of the non-targets. Jitter from the center of the grid position was applied randomly to each item within the aforementioned distance constraints. The color of each non-target letter in the search display was randomly selected with the constraint that a color could be used for no more than five letters on a given trial. The target was rendered in a particular color on half of the trials within each block (50%) and a different color on the other half of trials (selected at random with replacement from among the nine other colors on each of the remaining 50% of trials; see Anderson, 2026). Trials were presented in a random order each block. The frequent target color was randomly selected from among the ten possible colors for each participant. The orientation of the target (top of the rotated “T” on the left or right) was randomly determined on each trial with the constraint that each of the two target orientations was used equally often within each block, and the orientation of the “L” distractors was randomly determined on each trial with the constraint that no one orientation was used more than once more than any other.

#### Procedure

Participants were given two dissolvable strips and informed that the strips might or not might contain caffeine. They were not informed as to whether the strips they received contained caffeine, although the experimenter unpackaged the strips before the experiment, placing them into a clear bag, and was aware of their contents (single-blind). Participants held the strips in their mouths until they dissolved and then waited five minutes before proceeding. Participants then completed ten practice trials of the search task for which the target was equally likely to be presented in any of the ten possible colors, followed by the completion of the experimental trials.

Each trial began with the fixation display for 1000 ms, followed by the search display. The search display would remain until a response was recorded, and response time (RT) was measured from the onset of the search display. Participants pressed the “n” key if the top of the sideways “T” was on the left and the “m” key if the top of the sideways “T” was on the right. The ITI (which included “Incorrect” feedback if the wrong key had been pressed) was presented for 1000 ms. A minimum 30 s break occurred between blocks of trials.

#### Data analysis

Only the RTs for correct trials were, submitted to analysis and RTs less than 200 ms or exceeding 3 standard deviations of the mean of a given participant were trimmed. Search slope and intercept were computed for each participant via linear regression using display set size as the independent variable and RT as the dependent variable. These were computed separately for trials on which the target was rendered in the frequent versus an infrequent color. We then performed a mixed-effects analysis of variance (ANOVA) with target color frequency (frequent vs. infrequent) as a within-subjects variable and participant group (caffeine vs. placebo) as a between-subjects variable. Bayes Factors for main effects and interactions were computed via JASP using default priors (r scale = 0.5 for fixed effects, 1.0 for random effects, and 0.354 for covariates) to aid in quantifying the probability of the null hypothesis in cases where caffeine had a non-significant effect. BF₀₁ refers to evidence in favor of the null hypothesis, BF_10_ as evidence in favor of the alternative hypothesis, and BF_M_ as evidence in favor of the model with the indicated factors, with values greater than three interpreted as moderate evidence in favor thereof. Mean response time across participants is plotted as a function of display set size, fit with a regression line, for the purposes of visualization.

### Results

**Fig. 2:**
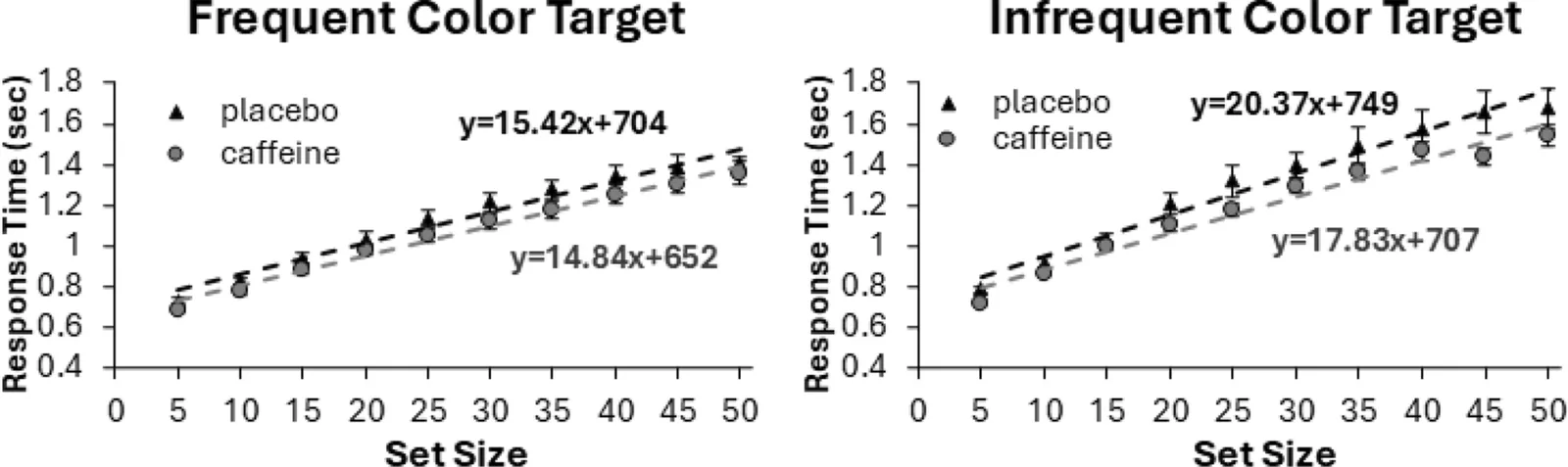
Response time as a function of set size and participant group (placebo, caffeine), separately for trials on which the target was rendered in the frequent and infrequent color in Experiment 1. Error bars represent the SEM

Mean accuracy was 98.7% in the caffeine group and 98.9% in the placebo group, which did not significantly differ, *t*(89) = 0.33, *p* = .746, BF₀₁ = 4.35. A 2 × 2 mixed-effects ANOVA on search slope with target color frequency (frequent vs. infrequent) as a within-subjects variable and participant group (caffeine vs. placebo) as a between-subjects variable revealed a main effect of target color, *F*(1,89) = 19.43, *p* < .001, ${\eta}_{p}^{2}$ =.179, while the main effect of participant group, *F*(1,89) = 0.91, *p* = .343, and the interaction,* F*(1,89) = 1.05, *p* = .308,^1^ were not significant (Fig. 2). A Bayesian ANOVA indicated that the data were best explained by a main effect of target color frequency only, BF_M_ = 7.21, with models including an additional main effect of group, BF_01_ = 2.48, and two main effects plus an interaction, BF_01_ = 6.73, being comparatively unlikely. When trial block was added as a within-subjects factor, there were no significant interactions between block and either of the other factors nor a three-way interaction (see Supplemental Material), providing no evidence for an effect of caffeine that only emerged late in the task.

The same ANOVA performed on search intercept revealed both a main effect of target color frequency, *F*(1,89) = 39.02, *p* < .001, ${\eta}_{p}^{2}$ = .305, and participant group, *F*(1,89) = 4.83, *p* = .031, ${\eta}_{p}^{2}$ =.051, but no interaction, *F*(1,89) = 0.39, *p* = .537.^2^ A Bayesian ANOVA indicated that the data were best explained by a model with a main effect of both target color frequency and group, BF_M_ = 5.20; a model with an interaction term added was comparatively unlikely, BF_01_ = 3.65. When trial block was added as a within-subjects factor, there was a significant interaction between block and group by which the effect of caffeine seemed to be maximal at later blocks (see Supplemental Material); no other interactions involving block were significant.

### Discussion

Participants were able to learn the color regularities of the visual search task and find and report the target more efficiently when it was rendered in the frequent target color, resulting in a reduced search slope and intercept. This reflects an influence of selection history on the control of attention and is consistent with the results of Anderson (2026). Only the intercept was modulated by acute administration of caffeine, which did not modulate (interact with) selection history-dependent effects observed in either search slope or intercept. While the main effect of participant group was not significant in the case of search slope, the difference between a model including this main effect and the preferred model with only a main effect of target color frequency was anecdotal (BF_01_ = 2.48). There was, however, moderate evidence against an interaction term between target color frequency and participant group for each dependent measure. Overall, we see evidence of caffeine affecting the speed with which observers can recognize a stimulus as a target and report it as such, but we do not see evidence of caffeine facilitating the speed with which non-targets can be rejected or the degree to which the attentional system can capitalize on learning-dependent processes in the guidance of goal-directed attentional orienting.

## Experiment 2

Experiment 2 investigated the effects of caffeine on a different aspect of attentional control, namely the ability to resist distraction by a salient but irrelevant stimulus along with the degree to which such distraction is modulated by feature-based selection history. Participants searched for a shape-defined target while trying to ignore a color singleton distractor that was presented on a subset of trials, completing the same task as the additional singleton task used in Youn and Anderson (2024), which was adapted from Stilwell et al. (2019). The physically salient distractor was more likely to be rendered in one particular color, which served as the high-probability distractor color. Previous research shows that distractors rendered in a high-probability color are more efficiently ignored as a result of selection history (Stilwell et al., 2019; Youn & Anderson, 2024), an effect of statistical learning that may operate through both proactive (Duncan et al., 2026; Richter et al., 2025) and reactive mechanisms of attentional control (Chen et al., 2026). Of interest was whether the cost in response time associated with the distractor, both in general and as a function of the statistical regularities of the task, could be modulated by acute caffeine administration.

### Method

#### Participants

86 new participants between the ages of 18 and 35 years inclusive, 43 in the caffeine group (25 females, 18 males, *M*_age_ = 18.86, SD = 1.10) and 43 in the placebo group (27 females, 15 males [1 not reported], *M*_age_ = 18.95, SD = 0.88), were recruited from the Texas A&M University community. Forty-five participants were again targeted for each group and more no-shows than budgeted resulted in two participants fewer per group in the final sample than was targeted. The inclusion and exclusion criteria, as well as the compensation for participation, were identical to Experiment 1. All procedures were approved by the Texas A&M Institutional Review Board. Written informed consent was obtained for each participant and all study procedures were conducted in accordance with the principles expressed in the Declaration of Helsinki.

#### Apparatus

Identical to Experiment 1.

#### Stimuli

**Fig. 3:**
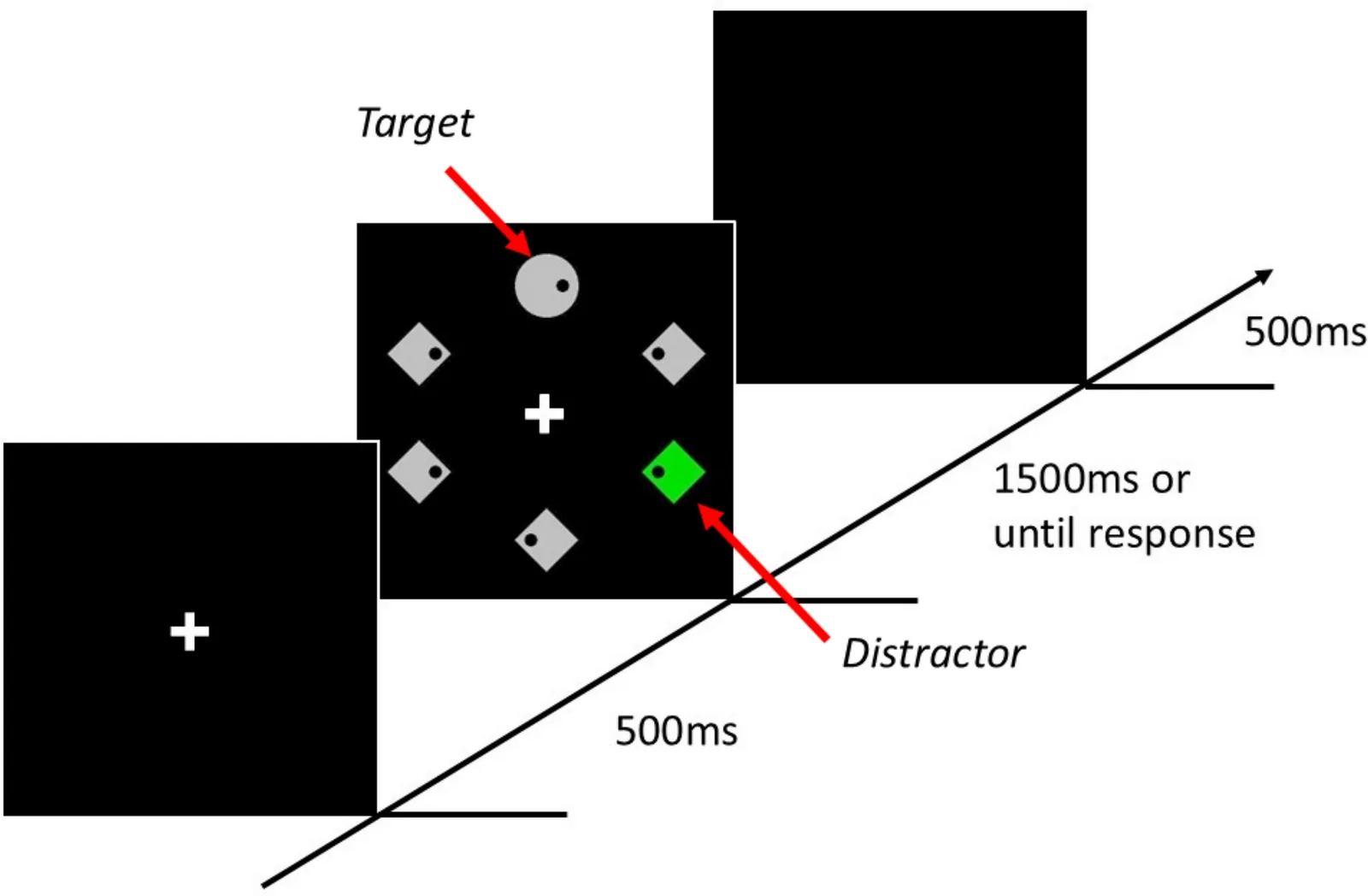
Example of a distractor-present trial with time course of events for Experiment 2

On each trial, six filled shapes (each 3.6° × 3.6° in size) were presented surrounding a white fixation cross at an equal distance on an imaginary circle with a radius of 8.25° (Fig. 3). Each shape contained a single black dot appearing on the left or right of the shape. The shapes presented were circles and diamonds, one of which was unique from the rest (i.e., five diamonds with one circle). On distractor-present trials, one of the non-targets was rendered in red, blue, or green; shapes were otherwise rendered in gray. After an initial fixation display (500 ms), the search array was presented for 1500 ms or until a response, followed by a blank inter-trial-interval (500 ms).

#### Design.

The experiment consisted of 1200 trials. During the experiment, one distractor color was more frequently presented (high-probability color, 50%) than the other two colors (low-probability colors, 20% combined [10% each]). Trials with no color singleton distractor were also presented (distractor-absent, 30%). A self-paced break occurred every 200 trials. Each color distractor was presented equally often at each location in the search array. The location of the dot within the shapes was randomly determined on each trial with the constraint that half of the dots appeared on the left and half on the right. Half of the trials were presented with five diamonds and one circle, and vice versa for the other half of trials. Trials were presented in a randomized order.

#### Procedure

Caffeine and placebo strips were administered in the same manner as Experiment 1. Five minutes after the caffeine administration, participants practiced the task (36 trials) where they were instructed to indicate the position of the dot inside the unique shape using the left and right arrow keys. Responses needed to be entered before the search array offset in order to be counted. Participants were informed that the color of the shapes was irrelevant to the task. An errant response or a failure to respond within the allotted 1500 ms resulted in corrective feedback (“Incorrect” or “Too Slow”) added after the search array for 1000 ms.

#### Data Analysis

Using the same RT trimming criteria as Experiment 1, mean RT for correct trials was computed for distractor-absent trials, trials containing a high-probability distractor, and trials containing a low-probability distractor for each participant. These means were then subjected to a 3 × 2 mixed-effects ANOVA with distractor condition (absent, high-probability, low-probability) as a within-subjects factor and participant group (caffeine, placebo) as a between-subjects factor. Where effects involving the within-subjects factor were significant, planned comparisons assessing the magnitude of attentional capture (both distractor conditions vs. absent) and the effect of selection history on attentional capture (high- vs. low-probability distractor conditions) were performed.

#### Results

**Fig. 4:**
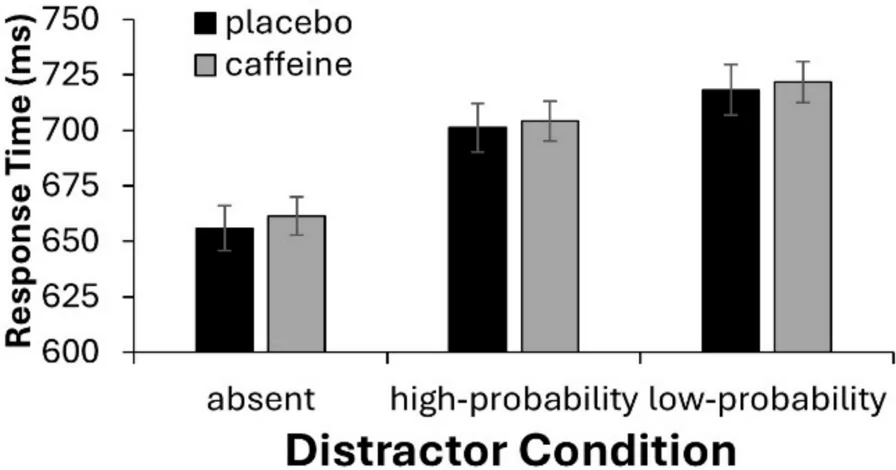
Response time as a function of distractor condition and participant group (placebo, control) in Experiment 2. Error bars represent the SEM

A mixed-effects ANOVA on RT with distractor condition (absent, high-probability, low-probability) as a within-subjects factor and participant group (caffeine, placebo) as a between-subjects factor revealed a significant main effect of distractor condition, *F*(2,168) = 476.42, *p* < .001, ${\eta}_{p}^{2}$ = .850 (see Fig. 4). Planned comparisons revealed that participants were distracted by the color singleton (distractor present vs. absent), *t*(85) = 24.34, *p* < .001, *d*_z_ = 2.62, and this distraction was attenuated for the high-probability compared to the low-probability distractor, *t*(85) = 12.06, *p* < .001, *d*_z_ = 1.30. The main effect of participant group, *F*(1,84) = 0.08, *p* = .776, and the interaction, *F*(2,168) = 0.20, *p* = .816,^3^ were not significant. A Bayesian ANOVA indicated that the data were best explained by a model with a main effect of distractor condition only, BF_M_ = 5.69; a model with an additional main effect of group, BF_01_ = 1.57, and two main effects plus an interaction, BF_01_ = 15.75, were comparatively less likely. When trial block was added as a within-subjects factor, there were no significant interactions between block and either of the other factors nor a three-way interaction (see Supplemental Material), providing no evidence for an effect of caffeine that only emerged late in the task.

Accuracy was generally high (*M* > 95%). The same ANOVA on accuracy revealed a significant main effect of distractor condition, *F*(2,168) = 37.09, *p* < .001, ${\eta}_{p}^{2}$ = .306 (see Table 1). As in RT, there was a performance cost associated with the distractor, *t*(85) = 8.05, *p* < .001, *d*_z_ = 0.87, however there was no difference between the high- and low-probability distractor conditions, *t*(85) = 0.87, *p* = .193. Neither was there a main effect of participant group, *F*(1,84) = 1.01, *p* = .317, nor an interaction, *F*(2,168) = 2.71, *p* = .069. A Bayesian ANOVA indicated that the data were again best explained by a model with a main effect of distractor condition only, BF_M_ = 4.14; a model with an additional main effect of group, BF_01_ = 1.77, and two main effects plus an interaction, BF_01_ = 2.50, were comparatively unlikely.

**Table 1:** Accuracy (SEM) as a function of distractor condition and participant group in Experiment 2

	Absent	High-probability	Low-probability
Placebo	96.3% (0.5%)	94.7% (0.6%)	94.9% (0.5%)
Caffeine	96.7% (0.3%)	95.8% (0.4%)	95.3% (0.5%)

### Discussion

The color singleton distractors robustly captured attention, the magnitude of which was attenuated for the high-probability distractor via selection history. Acute administration of caffeine did not modulate either of these effects, having no interaction with distractor condition. Neither was it the case that acute administration of caffeine generally influenced the speed of responses, although the difference between a model containing only a main effect of distractor condition and a model adding a main effect of participant group was anecdotal (BF_01_ = 1.57); a model in which caffeine modulated the magnitude of attentional capture, producing an interaction with distractor condition, could be more confidently rejected (BF_01_ = 15.75). In summary, we see no evidence for the acute administration of caffeine enhancing the ability to ignore distractors or learning to leverage statistical regularities when allocating attention. We did not detect a general speeding of motoric responding by caffeine either, although the evidence against such an effect was not conclusive.

## Experiment 3

Experiments 1 and 2 suggest a limited influence of caffeine on attentional performance, with a measurable influence only evident in the speed of target report under more demanding search conditions (Experiment 1). One possibility is that acute caffeine administration facilitated attentional control by making the task easier to complete without necessarily benefiting performance, empowering participants to exert less effort to achieve comparable levels of performance. A paradigm introduced by Anderson and Lee (2023, Experiment 5), which is an adaptation of the Adaptive Choice Visual Search (ACVS) task (Hansen et al., 2019; Irons & Leber, 2016, 2018; Kim et al., 2021Lee et al., 2022), provides a means of quantifying the subjective effort of searching. Specifically, two targets are presented on each trial, one red and another blue, and participants are free to find and report either one of them. The ratio of non-targets rendered in red and blue varies across trials, making it more difficult to search among items rendered in the more numerous color. Importantly, participants must exert physical effort using a hand dynamometer at the conclusion of each trial and reporting a target in one of the two colors results in a greater demand on physical effort than the other. Of particular interest are trials on which the more physically effort-demanding color is easier to find and report, creating a conflict between physical and attentional demands (see Anderson et al., 2025). To the degree that acute caffeine administration reduces the subjective effort of searching, we would expect participants to be more willing to search for and report the more difficult-to-find target, skewing the trade-off between physical and attentional effort away from attentional demands.

### Method

#### Participants

88 new participants between the ages of 18 and 35 years inclusive, 44 in the caffeine group (24 females, 19 males [1 not reported], *M*_age_ = 19.18, SD = 1.24) and 44 in the placebo group (19 females, 25 males, *M*_age_ = 18.96, SD = 0.96), were recruited from the Texas A&M University community. Forty-five participants were again targeted for each group and more no-shows than budgeted resulted in one participant fewer per group in the final sample than was targeted. The inclusion and exclusion criteria, as well as the compensation for participation, were identical to Experiment 1 and 2. All procedures were approved by the Texas A&M Institutional Review Board. Written informed consent was obtained for each participant, and all study procedures were conducted in accordance with the principles expressed in the Declaration of Helsinki.

#### Apparatus

Identical to Experiment 1 and 2 with the addition of a Vernier hand dynamometer (model HD-BTA) to measure physical effort.

#### Calibration

The calibration procedure mirrored Anderson and Lee (2023). Participants squeezed the hand dynamometer as hard as they could using their left hand over three trials to determine their individually calibrated grip strength. Each trial was separated by a five second rest period. On each trial, participants saw the text “Ready…” (1 s), “Set…” (1 s), and then “SQUEEZE!!” to signal when to apply force to the hand dynamometer. The word “SQUEEZE!!” remained on the screen for 3 s, and the output from the hand dynamometer was recorded over this entire 3 s duration. The individually calibrated force threshold for each participant was set at the median of nonzero values recorded from the device during the “SQUEEZE!!” epochs over the three trials.

#### Stimuli

**Fig. 5:**
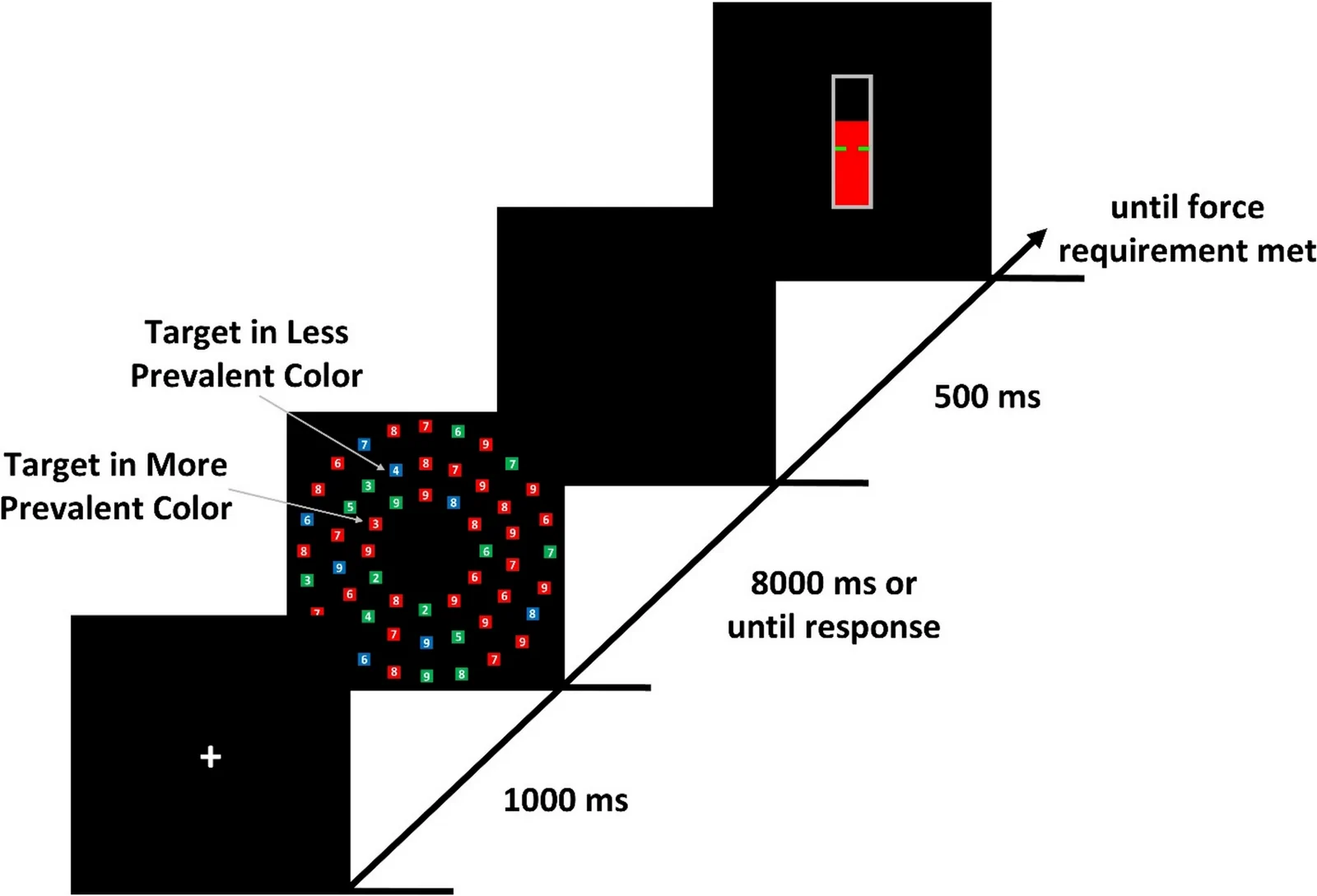
Example of trial sequence with time course of events for Experiment 3

Each trial began with a fixation display for 1000 ms (Fig. 5). A visual search array then appeared, where 54 colored squares (each approximately 1.1° × 1.1° in size) were arranged at equal intervals over three imaginary circles with the radius of 7.3°, 10.1°, and 13.0°. Color squares were presented in red, blue, and green, and each square had a white digit between 2 to 9, subtending 0.4° × 0.4°, presented within it. For red and blue squares, only one square for each color contained a digit between 2–5, while the other squares contained a digit 6–9; green squares could contain any digit between 2–9. After a response was indicated or 8000 ms elapsed, following a blank inter-stimulus-interval (ISI), a force meter appeared on the screen, which filled in proportion to the force applied to the hand dynamometer. A broken green line within the force meter indicated the target fill/force for that trial. Each trial concluded with a blank inter-trial-interval (ITI).

#### Design

The experiment consisted of four blocks of 60 trials each. Different color distributions were present in each block, where there were 10 trials each for the red-blue square ratios of 3:37, 8:32, 13:27, 27:13, 32:8, and 37:3. The remainder of the squares were always green (14 squares). The position of the color squares and the numbers within them were determined randomly, with the exception that only one red and one blue square contained a number 2–5 and this number was always different between the two (such that which number participants reported was diagnostic of which target they had found). One color was determined to be the more physically demanding color that would trigger a greater physical effort requirement to progress to the next trial, which alternated between red and blue every other participant.

#### Procedure

Caffeine and placebo strips were administered in the same manner as Experiments 1 and 2. Five minutes after the caffeine administration, participants were calibrated on the hand dynamometer. A practice task (32 trials) was then completed as participants were provided detailed task instruction, with the timeout limit and force requirement gradually introduced throughout practice. Participants were instructed to press the “v,” “b,” “n,” and “m,” keys on the keyboard using their right index, middle, ring, and pinky fingers which mapped onto target digits 2–5, respectively. They were explicitly informed that reporting one of the two color targets would trigger an “easy” grip requirement to progress to the next trial, while reporting the other color target would trigger a more difficult grip requirement, which would always be the case (100% predictive). They were also informed that there would be more red than blue items or vice versa on each trial, making it easier to search through the color of which there were fewer items, and that this would sometimes create a conflict between the target that would be easier to find and the target that would result in the easiest physical effort requirement. Participants were asked to search in whatever manner they thought was best for them.

Performance feedback was provided in the event that an invalid response as provided (a digit was reported that did not correspond to either target) or the search display had timed out, which was presented centrally for 1000 ms. The ISI was 500 ms and the ITI was 1000 ms in duration. The “easy” force requirement consisted of the need to apply 10% of calibrated maximal force for 200 ms, while the more difficult force requirement consisted of the need to apply 40% of calibrated maximal force for 3 s. The color of the fill of the force meter matched the color of the target reported. In the event of a timeout or incorrect response, the force meter filled white with force applied and the force requirement was always the more difficult one.

#### Data analysis

The primary dependent measure was the proportion of targets reported in the less-prevalent color as a function of the physical effort demands associated with that color, which was subjected to a 3 × 2 × 2 mixed-effects ANOVA with number of squares in the less-prevalent color (3, 8, 13), the physical effort associated with the less-prevalent color (high, low), and participant group (caffeine, placebo) as factors.

### Results

**Fig. 6:**
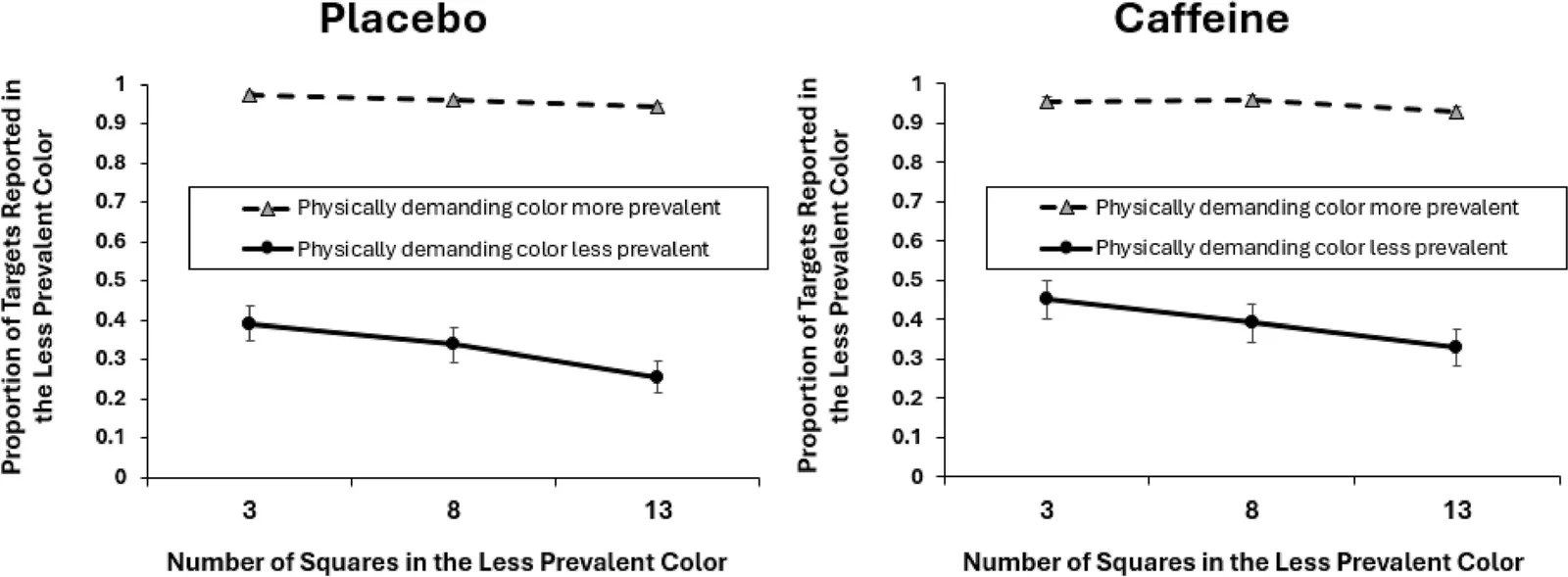
Proportion of targets reported in the less-prevalent (easier to search through) color as a function of participant group (placebo, caffeine), number of squares in the less-prevalent color, and which color was associated with greater physical demand in Experiment 3. Error bars represent the SEM

A 3 ×2 × 2 mixed-effects ANOVA with color distribution (number of squares in the less-prevalent color: 3, 8, 13) and physical demand (whether the less-prevalent color was associated with high or low effort) as within-subjects factors and participant group (caffeine, placebo) as a between-subjects factor revealed a significant main effect of color distribution, *F*(2, 172) = 78.33, *p* < .001, ${\eta}_{p}^{2}$ = .477, and physical demand, *F*(1, 86) = 296.43, *p* < .001, ${\eta}_{p}^{2}$ = .775 (Fig. 6). The interaction between color distribution and physical demand was also significant, *F*(2, 172) = 29.33, *p* < .001, ${\eta}_{p}^{2}$ = .254, which directly replicates Anderson and Lee (2023). However, no significant main effect of group was observed, *F*(1, 86) = 0.73, *p* = .397, nor did group interact with color distribution, *F*(2, 172) = 0.33, *p* = .719, or physical demand, *F*(2, 172) = 0.86, *p* = .427.^4^ Lastly, the three-way interaction was not significant, *F*(2, 172) = 0.45, *p* = .640. A Bayesian ANOVA indicated that the data were best explained by a model with a main effect of color distribution, physical demand, and an interaction between them, BF_M_ = 1679.85, with all other models being comparatively unlikely, BFs_01_ > 100. When trial block was added as a within-subjects effect, there was no significant interaction between block and group nor any other interactions involving block and group (see Supplemental Material), providing no evidence for an effect of caffeine that only emerged late in the task. The overall accuracy for both groups was 96.9%, with no difference between them, *t*(86) = 0.08, *p* = .938, BF_01_ = 4.47.

### Discussion

In Experiment 3, both attention and physical effort had a significant effect on how participants searched. When the easier-to-find target was also associated with low physical effort demands, participants unsurprisingly selected this target the overwhelming majority of the time. When attentional and physical demands conflicted, however, participants were sensitive to variation in attentional demand, increasingly reporting the target associated with greater physical demand as it became increasingly easier to find, resulting in a significant interaction between the color distribution of stimuli and physical demand. Importantly, these preferences were not shifted by acute caffeine administration. Had caffeine made the attentional demands of searching subjectively easier, we would have expected participants to be more willing to search for the harder-to-find target, particularly when motivated by physical effort demands to do so. This is not what we observed, with no evidence for any interactions between caffeine administration and either attentional or physical demands on effort.

It could be argued that caffeine also made the task of exerting physical effort subjectively easier, to a degree that offset any changes in the subjective effort of searching, essentially making the entirety of the task subjectively easier. The present experiment is unable to distinguish between offsetting influences and no influence of caffeine, which is intrinsic to a situation in which two kinds of demands on effort trade-off. It is clear, however, that attentional demands are not differently impacted by caffeine, which is inconsistent with the idea that caffeine selectively boosts the ability to focus attention, in this case in the context of voluntary attentional control during visual search.

## General discussion

Acute administration of caffeine has been shown to have selective beneficial effects on attention (Brunyé et al., 2010a, 2010b; Lorist et al., 1994, 1996) and learning (Borota et al., 2014; Nehlig, 2010). In the present study, we tested whether the guidance of voluntary attention during visual search, the ability to resist involuntary distraction, the learning-dependent modulation of each of these attentional processes, and the subjective demand of exercising attentional control in the context of visual search can be enhanced with caffeine. In doing so, we sought to expand the breadth with which attentional processes have been related to caffeine intake.

Across three experiments, we employed visual search tasks to examine the effects of acute administration of caffeine on the control of attention. The findings indicated that these effects were limited to processes related to the identification and reporting of the target during a demanding goal-directed visual search task. Caffeine did not improve the speed with which non-targets could be rejected, either in the context of a demanding visual search task (Experiment 1) or in the context of overcoming involuntary attentional capture by a difficult-to-ignore distractor (Experiment 2). Caffeine also did not render participants more willing to accept more difficult attentional demands in exchange for reduced physical workload (Experiment 3). When the target of visual search needed to be verified upon selection in a difficult visual search task that involved sequentially selecting stimuli (serial search; see Duncan & Humphreys, 1989; Wolfe, 2020, 2021), a beneficial effect of caffeine was evident (Experiment 1), which was not observed in a task in which the target was discernable on the basis of its feature contrast and easily discriminable from non-targets (Experiment 2). Coupled with more robust effects of caffeine on executive attention compared to the orienting of attention (Brunyé et al., 2010a, 2010b), our findings suggest that previously observed effects of caffeine on attention (e.g., Lorist et al., 1994, 1996) may be more strongly mediated by the efficiency with which task-relevant information is processed once selected (e.g., as observed in the Attention Networks Test and Stroop task) and less about the guidance of spatial attentional orienting, the latter of which was consistently unsupported in the present study.

Although acute administration of caffeine has been shown to improve incidental learning under certain conditions (Nehlig, 2010), we did not see evidence for caffeine improving the ability to learn from and leverage feature-based regularities either in the context of target guidance (Experiment 1) or distractor rejection (Experiment 2). Color-based regularities had a robust influence on performance in each of these experiments, demonstrating that participants learned the contingencies, but learning-dependent benefits in task performance were not further improved with caffeine. It is possible that any influence of caffeine was hampered by a ceiling effect, with the observed learning-dependent benefits being too robust to improve upon, which reflects a limitation of the present study. More subtle statistical regularities, with a more muted impact on the control of attention during visual search, may be more susceptible to modulation by caffeine. At least under conditions in which there are predictable regularities with which certain stimuli can be anticipated with high-probability, it does not appear to be the case that moderate doses of caffeine can enhance a person’s ability to take advantage of these robust regularities to the benefit of performance.

Our findings should be interpreted in the context of some additional specific limitations. 200 mg of caffeine was administered, which is consistent with multiple prior studies that have produced significant effects of caffeine (e.g., Borota et al., 2014; Brunyé et al., 2010b; Lorist et al., 1994; note that the also commonly used 3 mg/kg dose is approximately equivalent to 200 mg). However, it is possible that more robust effects would have been evident at a higher dose. The placebo strips, while containing no caffeine, do contain active ingredients such as menthol that could have some transient alerting properties and so do not constitute an entirely neutral baseline.

Although participants in our study self-reported abstaining from caffeine intake at least four hours prior to study participation, this was not objectively verified. Additionally, although we screened out heavy users of caffeine, participants likely differed to some degree with respect to their typical daily caffeine intake. We did not collect information about participants’ normal daily intake of caffeine beyond our screening criteria. More robust effects might have been observed for more habitual users of caffeine who are caffeine-deprived, or possibly for individuals who infrequently use caffeine and for whom caffeine might have a more pronounced impact. Similarly, the effects of caffeine may be greater when tested in a sleep-deprived state. We were, however, interested in the potential for caffeine to enhance attentional processes in a moderate dose and not in the ability of caffeine administration to mitigate withdrawal-related effects or the detrimental consequences of sleep deprivation. In this context, the use of caffeine as an everyday cognitive enhancer appears to have limited benefits with respect to the components of attention measured, although benefits in specific subgroups of users (e.g., heavier users) may be more pronounced.

The cognitive effects of caffeine are dependent on the pharmacokinetic effects of the substance. We used caffeine strips, absorbed sublingually via the oral mucosa, to maximize the efficiency with which the effects of caffeine would be realized. The impact of substances administered sublingually is generally realized within a few minutes (e.g., Narang & Sharma, 2011), which is consistent with manufacturer specifications for the caffeine product we used and our brief five-minute delay before testing. Nevertheless, the time course with which administered caffeine might impact cognitive processes inclusive of attention is unclear. Adding trial block to analyses did not produce evidence of time-dependent interactions, which would be expected for an effect of caffeine that only emerged later in the course of the task, with the exception of the effect of group (caffeine vs. placebo) on search intercept in Experiment 1, which was already significant when collapsed over all trials. That our only significant effect of caffeine resulted in a significant interaction with block suggests some sensitivity to detect such interactions and is inconsistent with a more robust effect of caffeine for other experiments and measures that was only detectable later in the task. However, the possibility that caffeine may have a more pronounced impact when tested at a later point in time following administration cannot be ruled out.

Participants were blinded to whether the orally dissolvable strips they were administered contained caffeine. However, the experimenter who unpackaged the strips was aware of their contents, and more broadly, it is possible that participants had suspicions concerning the condition to which they were assigned based on the flavor of the strips and its presumed relationship to caffeine in commercially available products. We did not query participants concerning their perceptions of the dissolvable strips they were administered and whether they thought they contained caffeine. However, to the degree that participants had suspicions concerning the condition to which they were assigned and these suspicions produced a demand characteristic, we would have expected more robust effects of caffeine, which had a very limited observable influence on performance in the present study. It therefore seems unlikely that the results of the present study were the product of a demand characteristic, although the single-blind nature of the study and the sensory differences between the caffeine and placebo strips more broadly complicate interpretation of the findings.

Overall, our findings cast doubt on the idea that caffeine, at least in moderate doses, generally promotes processes of selective attention and/or attentional learning in the context of visual search. Participants were no more efficient in filtering non-targets, resisting distraction by salient stimuli, and leveraging statistical regularities in the context of either target guidance or distractor rejection. They did not prioritize attentional and physical effort any differently than participants who were not administered caffeine. The only significant effect of caffeine observed in the present study was with respect to search intercept in Experiment 1, which is more difficult to interpret, as it could reflect any component process for which the impact of caffeine does not scale with set size. This intercept difference could reflect the speed with which a match between the target and a target template (Woodman et al., 2013) is made post-selection, the pre-selection build-up of the attentional priority map (Lleras et al., 2020; Wolfe, 2021), or attentional readiness (Iemi & Busch, 2018; Ruzzoli et al., 2019) and the speed with which attentional processing is initiated upon display onset. It could also simply reflect faster manual responding, which if true would have also been expected in Experiment 2, with the data providing only equivocal support for the null hypothesis. On the whole, when it comes to professions that involve high attentional demand in the context of visual search, such as radiology, air traffic control, and security/surveillance, our findings argue against the idea that caffeine can be used to provide a temporary boost to performance, especially when considered in the context of cognitive enhancement (as opposed to, for example, masking the effects of sleep deprivation, which was not examined in the present study).

## Data Availability

Datasets are available from the corresponding author on reasonable requests.

## Footnotes

1 From the measured effect sizes, a sample size of *n* = 652 would be needed to achieve power = 80% to detect a main effect of group, and *n* = 108 to detect an interaction.

2 From the measured effect size, a sample size of *n* = 266 would be needed to achieve power = 80% to detect an interaction.

3 From the measured effect sizes, a sample size of *n* = 7868 would be needed to achieve power = 80% to detect a main effect of group, and *n* = 68 to detect an interaction.

4 From the measured effect sizes, a sample size of *n* = 330 would be needed to achieve power = 80% to detect a main effect of group, *n* = 180 to detect an interaction between group and physical demand, and *n* = 36 to detect an interaction between group and color distribution.

## Declarations

### Human and animals rights

All procedures were approved by the Texas A & M Institutional Review Board and performed in accordance with the principles outlined in the Declaration of Helsinki.
